# The Role of Immune Checkpoint Inhibitors in the Treatment of Extensive-Stage Extrapulmonary Small Cell Carcinoma

**DOI:** 10.7759/cureus.8862

**Published:** 2020-06-27

**Authors:** Ragia Aly, Sachin Gupta, Rashmika Potdar

**Affiliations:** 1 Internal Medicine, Danbury Hospital, Danbury, USA; 2 Internal Medicine, Reading Hospital, West Reading, USA; 3 Hematology and Medical Oncology, Geisinger Medical Center, Danville, USA

**Keywords:** immune-checkpoint inhibitors, extrapulmonary, small cell carcinoma, small-cell lung carcinoma, immunotherapy, small cell

## Abstract

Small cell carcinoma is a type of highly aggressive poorly differentiated neuroendocrine tumor that can arise from multiple organs, including but not limited to bronchial tissue, pancreas, gastrointestinal tract, and genitourinary system. The most commonly studied type is small cell lung cancer (SCLC) which carries the worst prognosis among lung cancers. After multiple promising clinical trials, the National Comprehensive Cancer Network has recently added atezolizumab and durvalumab in combination with platinum-based chemotherapy/etoposide to the first-line treatment regimen for extensive-stage SCLC (ES-SCLC). Meanwhile, the recommended treatment for extrapulmonary small cell carcinoma (EPSCC) remains unchanged. In this review, we try to explore the role of immunotherapy in the treatment of EPSCC.

## Introduction and background

Small cell carcinomas are poorly differentiated highly aggressive neuroendocrine tumors that can arise from a variety of epithelial tissues, with the most common being the bronchial tree, constituting up to 15% of lung cancers [1]. Primary small cell carcinomas less commonly originate from the gastrointestinal tract, genitourinary system, skin, bone, and the pericardium [2,3]. Extrapulmonary small cell carcinoma (EPSCC) are very rare but highly aggressive tumors, with most cases presenting with widespread metastatic disease. Only 5% of diagnosed small cell carcinomas are extrapulmonary, with incidence rate ranging between 0.1% to 0.4% [4]. EPSCC is classified according to the Veterans Administration Lung Group (VALSG) to a limited stage with a median survival of 1.4 to 3.5 years and an extensive stage with a median survival of less than one year [5]. The staging for EPSCC is similar to that of small cell lung cancer (SCLC), and the median survival is comparable [1].

New and promising advances in the field of cancer immunotherapy have changed the management of multiple types of solid tumors, including SCLC, particularly immune checkpoint inhibitors, as they have shown great promise in multiple recent clinical trials, such as the CASPIAN trial (A Phase III, Randomized, Multicenter, Open-Label, Comparative Study to Determine the Efficacy of Durvalumab or Durvalumab and Tremelimumab in Combination With Platinum-Based Chemotherapy for the First-Line Treatment in Patients With Extensive Disease Small-Cell Lung Cancer) that investigated the effect of adding durvalumab to the standard therapy in patients with extensive-stage SCLC (ES-SCLC) [6], the IMpower133 trial (A Phase I/III, Randomized, Double-Blind, Placebo-Controlled Study of Carboplatin Plus Etoposide With or Without Atezolizumab (Anti-PD-L1 Antibody) in Patients With Untreated Extensive-Stage Small Cell Lung Cancer) that studied the use of atezolizumab in ES-SCLC as well [7], the KeyNote trials (A Randomized Open-Label Phase III Trial of MK-3475 Versus Platinum Based Chemotherapy in 1L Subjects With PD-L1 Strong Metastatic Non-Small Cell Lung Cancer) investigating pembrolizumab in combination with chemotherapy for treatment of different types of cancers [8-10] and the CheckMate 032 trial (A Phase 3, Randomized, Double-Blind Study of Nivolumab Monotherapy or Nivolumab Combined With Ipilimumab Versus Ipilimumab Monotherapy in Subjects With Previously Untreated Unresectable or Metastatic Melanoma) evaluating the anti-tumor activity of nivolumab, that opened the door to several subsequent CheckMate trials [11].

## Review

Cancer cells have the ability to evade the immune system by expressing fewer antigens on their surfaces or by downregulating the MHC class I expression [12]. More importantly, malignant cells can also defend themselves from being attacked by T-cells through expressing what is called immune checkpoint molecules. These molecules are part of a normal protective mechanism and are upregulated in response to the cytokines produced by the activated T-cells. This feedback mechanism is present to protect tissues from excessive inflammation through suppressing T-cells [13]. This mechanism has become the target of the newly developed Immune checkpoint inhibitors. Working on the inhibitory receptor/ligand combinations between T-cells and tumor cells, such as the programmed cell death protein-1 (PD-1) and programmed cell death-ligand 1 (PD-L1), and the cytotoxic T lymphocyte antigen-4 (CTLA-4/B7) [14,15]. CTLA4 counteracts the activity of the T cell costimulatory receptor CD28 for binding to the B7 ligand expressed on tumor cells and, therefore, CTLA4 functions as a negative immune regulator. Similarly, the linkage between PD-1 on T cells and the PD-L1 on the tumor cell surface inhibits T-cell proliferation, resulting in T cell function inhibition [16]. Multiple monoclonal antibodies targeting PD-1 and PD-L1, as well as CTLA-4, have recently been approved by the Food and Drug Administration (FDA) for the treatment of several malignancies, including SCLC, non-SCLC, renal cell cancer, urothelial cancer, melanoma and lymphoma [17], These FDA approved monoclonal antibodies started with the ipilimumab (human IgG1 k anti-CTLA-4 monoclonal antibody) in 2011, followed by the PD-1 inhibitors nivolumab, pembrolizumab, cemiplimab, and the PD-L1 inhibitors atezolizumab, avelumab, and durvalumab [18].

Among all small cell carcinomas, SCLC, being the most common, has been more extensively studied and was the target of two large clinical trials that have led to revolutionary changes in the first-line treatment guidelines for ES-SCLC after decades of stagnation [6,7]. Atezolizumab [19], an immune checkpoint inhibitor that blocks the interaction of PD-L1 with PD-1 and CD80 receptors (B7-1Rs) [20]. And more recently durvalumab [21], a selective, high affinity human IgG1 monoclonal antibody which impedes PD-L1 from binding to PD-1 and CD80 [22], to be used in combination with standard-of-care chemotherapy, etoposide, and platinum-based chemotherapy agent, as a frontline treatment for adult patients with ES-SCLC [6,7]. Meanwhile, there has not been any new advances in the treatment of EPSCC. The current National Comprehensive Cancer Network guidelines list multiple combination chemotherapy options for the treatment of poorly differentiated EPSCCs including cisplatin/etoposide and carboplatin/etoposide as first-line options. FOLFOX, FOLFIRI, and temozolomide with or without capecitabine are also suggested. Judging by the shared histologic origin, similar pathophysiology, and comparable clinical course between EPSCC and SCLC, is there a role for immune check point inhibitors in the treatment of EPSCC?

To our knowledge, the treatment recommendations for EPSCC have been derived from single-institution experiences or extrapolated from studies of SCLC [23], up until the recent approval of atezolizumab or durvalumab in combination with a platinum agent/etoposide combination as a preferred first-line regimen for ES-SCLC, the NCCN listed similar first-line treatment for ES-SCLC and ES-EPSCC, namely, cisplatin or carboplatin in combination with etoposide. In a retrospective study from Kansas University that looked into the treatment regimen and clinical response of 35 patients with primary EPSCC, 11 originated from the genitourinary tract, including the bladder, kidney, ureter, prostate, and urethra. Ten originated from various locations in the GI tract including the colon, rectum, pancreas, and esophagus. Six from the female reproductive organs, including the cervix, ovary, and Bartholin’s glands. Five were considered head and neck primary tumors from the base of the tongue, nasopharynx, maxillary sinus, thyroid, and parotid. Three cases were deemed to be from an unknown primary source. Forty-three percent of patients had metastatic disease at the time of diagnosis, the most commonly used chemotherapy regimens included etoposide combined with either cisplatin or carboplatin in 74% of the cases. The study revealed a dire five-month median survival for patients with extensive disease [24]. In a similar retrospective study that analyzed the data of 120 patients with a diagnosis of EPSCC with variable origins, 30% of patients had extensive disease, with a median survival of 0.7 years [23].

Role of PD-L1

In the recent clinical trial OAK that was conducted on 1225 patients with advanced non-squamous non-SCLC comparing atezolizumab to docetaxel revealed that the response rate to atezolizumab was better in patients whose tumors expressed higher PD-L1 levels. However, patients who had tumors with below median PD-L1 expression still benefited from treatment when compared to docetaxel. Moreover, overall survival improvement was noted in all PD-L1 expression subgroups, including those with low or undetectable PD-L1 [25].

In a clinical retrospective study done at the University of Massachusetts Medical cancer center on 34 patients with EPSCC who had sufficient tissue specimens for PD-L1 immunohistochemistry analysis, patients who had limited disease the overall response rate in the PD-L1 positive group was 80% versus 67% in the PD-L1 negative group. The median overall survival for the PD-L1 positive group, regardless of stage, was 11.5 months versus seven months for the PD-L1 negative group. Patients with limited-stage disease with positive PD-L1 staining had a median 53 months overall survival compared to PD-L1 negative patients with limited-stage disease whose median overall survival was 15 months. Extensive stage patients had median overall survival that was similar between PD-L1 positive and negative groups, four months vs five months respectively, which was not statistically significant [26].

Few case reports were published showing good response to immune checkpoints inhibitors in patients with ES-EPSCC. In a case of metastatic small cell carcinoma of the cervix in a 38-year-old female who had failed treatment with chemotherapy was treated with nivolumab, which is an immune checkpoint inhibitor that is specific for the PD-1 receptor. Despite the fact that her tumor showed absent PD-L1 expression, Radiographic imaging demonstrated a decrease in the size of all target lesions after two doses of treatment [27]. Another case reported a good response to nivolumab in a patient with metastatic small cell carcinoma of the pancreas after failing the first-line treatment with carboplatin and etoposide and failing treatment with topotecan. Her scans showed a significant reduction in tumor sizes after four doses of nivolumab [28].

Ongoing clinical trials on EPSCC

There are currently multiple ongoing clinical trials evaluating the effectiveness of immune checkpoint inhibitors in patients with EPSCC mostly involving the male genitourinary tract, the results of these trials are yet to be published. The active Phase II trial (NCT03866382) that started on April, 12,2019 is testing the effectiveness of two immunotherapy drugs (nivolumab and ipilimumab) with cabozantinib in patients with rare metastatic genitourinary tumors including small cell carcinoma of the bladder, this trial is expected to be completed in February 2023. Another clinical trial (NCT03910660) looking into the use of the new investigational drug BXCL701 which is an orally-available systemic innate immunity activator, in combination with pembrolizumab for treatment of metastatic small cell carcinoma of the prostate, this study is expected to conclude in April 2022. A third phase Ib trial (NCT03582475) studies how well pembrolizumab works with combination chemotherapy in treating participants with small cell/neuroendocrine cancers of the urothelium or prostate that have locally progressed, or metastasized to nearby lymph nodes or distant organs, this study is expected to report primary results in September 2020. To our knowledge, there are currently no other trials involving EPSCC of other tissue origins.

## Conclusions

The small number of EPSCC cases and the wide variety in their tissue of origin creates a significant challenge for designing prospective clinical trials. Conducting meaningful clinical trials require a large cohort of cases that should ideally have the same tissue of origin; this would require coordination with a large number of centers worldwide. And might take decades. The mainstay of treatment of small cell carcinomas that are not pulmonary in origin remains to be surgical resection, chemotherapy, radiation therapy, and/or neoadjuvant chemotherapy in limited-stage disease and chemotherapy in extensive-stage disease. With the promising data from clinical trials on SCLCs of pulmonary origin, and while we await the outcomes of new trials targeting EPSCC, we have to ask, should the immune checkpoints inhibitors that were approved for treating SCLC be used for the treatment of EPSCC until more data becomes available.

## References

[1] Travis WD (2012). Update on small cell carcinoma and its differentiation from squamous cell carcinoma and other non-small cell carcinomas. Mod Pathol.

[2] Raina V, Milroy R, al-Dawoud A, Dunlop D, Soukop M (1992). Extrapulmonary small cell carcinoma of bone. Postgrad Med J.

[3] Mohandas KM, Chinoy RF, Merchant NH, Lotliker RG, Desai PB (1992). Malignant small cell tumour (Askin-Rosai) of the pericardium. Postgrad Med J.

[4] Wong YNS, Jack RH, Mak V, Henrik M, Davies EA (2009). The epidemiology and survival of extrapulmonary small cell carcinoma in South East England, 1970-2004. BMC Cancer.

[5] Ochsenreither S, Marnitz-Schultze S, Schneider A (2009). Extrapulmonary small cell carcinoma (EPSCC): 10 years’ multi-disciplinary experience at Charité. Anticancer Res.

[6] Paz-Ares L, Dvorkin M, Chen Y (2019). Durvalumab plus platinum-etoposide versus platinum-etoposide in first-line treatment of extensive-stage small-cell lung cancer (CASPIAN): a randomised, controlled, open-label, phase 3 trial. Lancet.

[7] Horn L, Mansfield AS, Szczęsna A (2018). First-line atezolizumab plus chemotherapy in extensive-stage small-cell lung cancer. N Engl J Med.

[8] Fuchs CS, Doi T, Jang RW (2018). Safety and efficacy of pembrolizumab monotherapy in patients with previously treated advanced gastric and gastroesophageal junction cancer: phase 2 clinical KEYNOTE-059 trial. JAMA Oncol.

[9] Langer CJ, Gadgeel SM, Borghaei H (2016). Carboplatin and pemetrexed with or without pembrolizumab for advanced, non-squamous non-small-cell lung cancer: a randomised, phase 2 cohort of the open-label KEYNOTE-021 study. Lancet Oncol.

[10] Kato K, Shah MA, Enzinger P (2019). KEYNOTE-590: Phase III study of first-line chemotherapy with or without pembrolizumab for advanced esophageal cancer. Future Oncol.

[11] Ready N, Farago AF, de Braud F (2019). Third-line nivolumab monotherapy in recurrent SCLC: CheckMate 032. J Thorac Oncol.

[12] Tsukahara T, Kawaguchi S, Torigoe T (2006). Prognostic significance of HLA class I expression in osteosarcoma defined by anti-pan HLA class I monoclonal antibody, EMR8-5. Cancer Sci.

[13] Sharma P, Hu-Lieskovan S, Wargo JA, Ribas A (2017). Primary adaptive, and acquired resistance to cancer immunotherapy. Cell.

[14] Vesely MD, Kershaw MH, Schreiber RD, Smyth MJ (2011). Natural innate and adaptive immunity to cancer. Annu Rev Immunol.

[15] Chen DS, Mellman I (2013). Oncology meets immunology: the cancer-immunity cycle. Immunity.

[16] Freeman GJ, Long AJ, Iwai Y (2000). Engagement of the PD-1 immunoinhibitory receptor by a novel B7 family member leads to negative regulation of lymphocyte activation. J Exp Med.

[17] Arora E, Masab M, Mittar P, Jindal V, Gupta S, Dourado C (2018). Role of immune checkpoint inhibitors in advanced or recurrent endometrial cancer. Cureus.

[18] Vaddepally RK, Kharel P, Pandey R, Garje R, Chandra AB (2020). Review of indications of FDA-approved immune checkpoint inhibitors per NCCN guidelines with the level of evidence. Cancers.

[19] (2020). FDA approves atezolizumab for extensive-stage small cell lung cancer. U.S.

[20] Syn NL, Teng MWL, Mok TSK, Soo RA (2017). De-novo and acquired resistance to immune checkpoint targeting. Lancet Oncol.

[21] (2020). IMFINZI (durvalumab) approved in the US for extensive-stage small cell lung cancer. Wilmington, DE: AstraZeneca; March 30.

[22] Stewart R, Morrow M, Hammond SA (2015). Identification and characterization of MEDI4736, an antagonistic anti-PD-L1 monoclonal antibody. Cancer Immunol Res.

[23] Brennan S, Gregory D, Stillie A, Herschtal A, Manus MM, Ball DL (2010). Should extrapulmonary small cell cancer be managed like small cell lung cancer?. Cancer.

[24] Dakhil CS, Wick JA, Kumar AK, Satyan MT, Neupane P (2014). Extrapulmonary small cell carcinoma: the University of Kansas experience and review of literature. Med Oncol.

[25] Rittmeyer A, Barlesi F, Waterkamp D (2017). Atezolizumab versus docetaxel in patients with previously treated non-small-cell lung cancer (OAK): a phase 3, open-label, multicentre randomised controlled trial. Lancet.

[26] Salhab M, Migdady Y, Donahue M (2018). Immunohistochemical expression and prognostic value of PD-L1 in extrapulmonary small cell carcinoma: a single institution experience. J Immunotherapy Cancer.

[27] Paraghamian SE, Longoria TC, Eskander RN (2017). Metastatic small cell neuroendocrine carcinoma of the cervix treated with the PD-1 inhibitor, nivolumab: a case report. Gynecol Oncol Res Pract.

[28] Ugwu JK, Nwanyanwu C, Shelke AR (2017). Dramatic response of a metastatic primary small-cell carcinoma of the pancreas to a trial of immunotherapy with nivolumab: a case report. Case Rep Oncol.

